# Prokaryotic Diversity and Community Distribution in the Complex Hydrogeological System of the Añana Continental Saltern

**DOI:** 10.1007/s00248-025-02488-2

**Published:** 2025-01-16

**Authors:** Maia Azpiazu-Muniozguren, Minerva García-Martínez, Ane Zabaleta, Iñaki Antiguedad, Javier Garaizar, Lorena Laorden, Irati Martinez-Malaxetxebarria, Ilargi Martinez-Ballesteros

**Affiliations:** 1 MikroIker Research Group, Immunology, Microbiology and Parasitology Department, Faculty of Pharmacy, University of the Basque Country UPV/EHU, Paseo de La Universidad 7, 01006 Vitoria-Gasteiz, Spain; 2 Hydro-Environment Processes Research Group. Geology Department, Faculty of Science and Technology, University of the Basque Country UPV/EHU, Barrio Sarriena S/N, 48940 Leioa, Spain; 3 Bioaraba, Microbiology, Infectious Diseases, Antimicrobial Agents, and Gene Therapy, 01006 Vitoria-Gasteiz, Spain

**Keywords:** Salt diaper, Continental saltern, 16S rRNA gene sequencing, Prokaryotic diversity

## Abstract

The Añana Salt Valley (northern Spain) is a continental saltern consisting of a series of natural springs that have been used for salt production for at least 7000 years. This habitat has been relatively understudied; therefore, prokaryotic diversity was investigated through Illumina-based 16S rRNA gene sequencing to determine if the waters within the valley exhibit distinctive microbiological characteristics. Two main types of water were found in the valley: salty (approximately 200 g/L salinity) from the diapiric structure and brackish (≤ 20 g/L salinity) from shallow streams. The beta diversity indices showed that salinity was the primary factor influencing the prokaryotic distribution. However, a niche-specific influence was observed between waters of the same origin, with significant differences in the relative abundance of the ASVs. The microbiome of the saltern revealed that the archaeal domain was mainly restricted to salty waters, while the bacterial domain was ubiquitous throughout the saltern, with a notable prevalence in brackish waters. The main bacterial and archaeal phyla identified were *Pseudomonadota* and *Halobacterota*, respectively. The genus *Halorubrum* was abundant and widespread in salty waters, while *Pseudomonas* was a significant part of the prokaryote community, mainly in brackish waters. The relative abundance of the genera *Haloplanus* and *Salinibacter* increased in the salt ponds used for salt production. The taxa involved in chemoheterotrophy and fermentation were widespread, sharing the same niche. Overall, the location of this saltern on a diapiric structure favors the occurrence of waters with different origins that affect the prokaryotic distribution beyond the niche location in the valley.

**Supplementary Information:**

The online version contains supplementary material available at 10.1007/s00248-025-02488-2.

## Introduction

Hypersaline environments represent highly bioproductive habitats with a large microbial diversity adapted to these conditions [1]. To better understand these environments, it is essential to characterize the microbial communities due to their contribution to nutrient cycling and ecosystem functioning [2]. Therefore, the study of hypersaline environments such as solar salterns [3], salt mines [4], and salt lakes [5] has received considerable interest. Many different types of halophilic and halotolerant microorganisms including archaea of the order *Halobacteriales* and bacterial species of the order *Halanaerobiales* and *Halomonadaceae*, *Desulfohalobiaceae*, and *Salinibacteriaceae* families, among others [6], are found in these environments. While halophiles are less common within the Eucarya domain, the green alga *Dunaliella* is typically present in aerobic environments with high salt content [5]. As microbial communities are vital to ecosystem functions and are influenced by the physical and geological characteristics of the site, parallel studies in these other areas are also necessary to gain a comprehensive understanding of these ecosystems [1, 7].

Among the different types of salterns that exist, continental ones are less well known, and not many are still in use today. The Añana Salt Valley (Álava, Basque Country, Spain) is placed in one of the best-preserved continental salterns in Europe. Salt production is active in the valley since at least 7000 years, and it has been recognized by Food and Agriculture Organization (FAO) as being Europe’s first Globally Important Agricultural Heritage System (GIAHS). The underground evaporitic rocks, comprising salt, gypsum, clay and others, were formed during the initial stages of the fragmentation of Pangaea supercontinent (approximately 200 million years ago). These rocks now form a geologically complex diapiric structure where the salts are actively rising [8]. The geological complexity of the area leads to hydrogeological complexity, with very slow deep flows mixing with shallower flows in some areas of the valley. This results in the emergence of springs with varying salinity levels, dependent on the route taken by the water through the subsoil. This gives rise to the presence of springs with salty (≈ 200 g/L salinity) or brackish (≤ 20 g/L salinity) water that are very close to each other.

Despite the singularity of this environment, and although studies have been carried out in this saltern on archeology, hydrogeology, or the salt itself, there is limited microbiological data available. There is only one publication on microbial and viral changes from groundwater to surface water [9] and one on fungal community diversity [10], so further research is needed to fully understand this ecosystem. Therefore, in this context, this work present an extended study along this ancient saltern in which the prokaryotic diversity was studied by Illumina-based 16S rRNA gene sequencing to determine whether the waters within the valley have distinctive microbiological characteristics. Taking into account the physico-chemical parameters of the main waters supplying the valley (six springs, a stream that cross the saltern, and groundwater) and water involved in salt production (water from a brine distribution channel and three brine resting ponds), prokaryotic community structure and composition was studied. The integration of all data is not only important for expanding scientific understanding but also for facilitating the recovery and enhancement of this active saltern.

## Material and Methods

### Sampling Site Features and Sampling Procedure

**Fig. 1:**
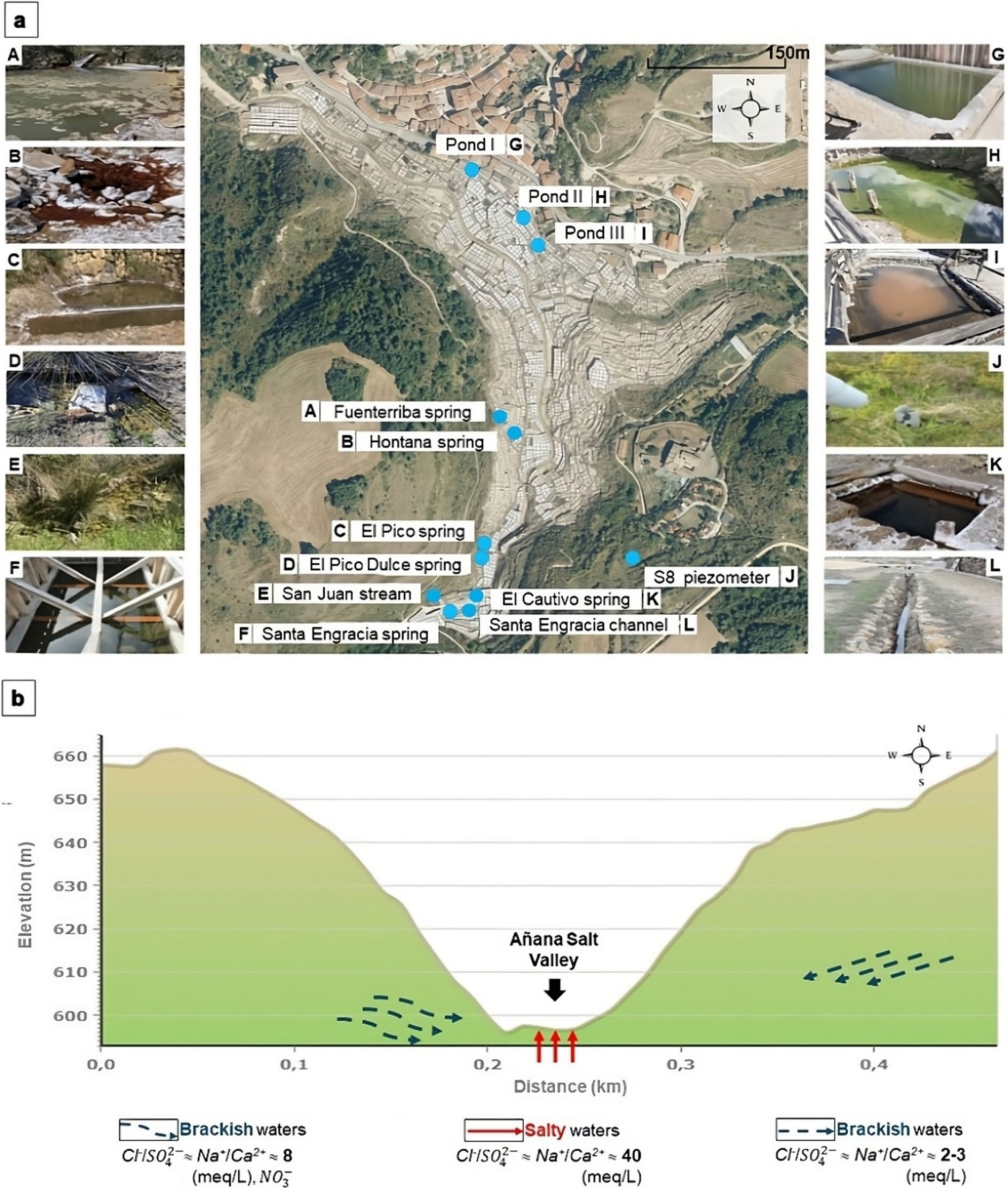
The Añana Salt Valley; **a** map showing the 12 sampling locations and their pictures (A-L) and **b** the representation of the different water flows in the valley

Añana Salt Valley (42°47′59.82″ N, 2°59′3.23″ W; altitude 570–598 m) is in western Álava, Basque Country, Spain. For this study, 12 sampling sites (Fig. 1a) were selected based on their accessibility and minimal human intervention. The Santa Engracia spring is situated at the highest point of the valley, and it is the main brine supplier to the saltern. Immediately, its water is channeled (called in this study Santa Engracia channel) and distributed throughout the valley to the temporary accumulation or resting ponds (i.e., Pond I, Pond II, and Pond III) from which the brine is distributed to the crystallization pans at the end of the salt production system. Santa Engracia spring is very close to the brackish water source of the San Juan stream, which runs all along the saltern, but it is not part of the salt production system. A little further north are the other springs, some salty (El Cautivo, El Pico, Fuenterriba, and Hontana) and some brackish (El Pico Dulce, which is only two meters from El Pico). To the east in the valley, there is a piezometer (called S8) from which brackish groundwater can be sampled at a depth of 60 m. Most of the springs are in direct contact with the natural surrounding environment, except for the Santa Engracia spring and the El Cautivo spring, which have a wooden structure around them (Fig. 1a). More information about the complex geological and hydrogeological context of the valley is presented in the Supplementary Material.

Given the importance of salt production in the economic and social development of the area and its relevance as a natural, cultural and heritage site, there is since 2017 a surface- and ground-water monitoring network throughout the valley. Of the 12 sampling sites, eight (the six springs, the stream, and the piezometer) are part of this monitoring network; therefore, the ionic composition of the water samples was available. This composition is periodically analyzed by ion chromatography at the SGIker Advanced Research Facilities of the University of the Basque Country UPV/EHU. In a first phase of this study (spring 2018), water samples from three springs (Santa Engracia spring, El Pico spring, and El Pico Dulce spring), groundwater from S8 piezometer, and water from the ponds (pond I, II, and III) were collected. In a second phase of the study (spring 2021), the sampling was extended to the rest of the springs (Fuenterriba spring, El Cautivo spring, and Hontana spring), the San Juan stream, and the brine distribution channel (Santa Engracia channel).

Water samples were collected directly using sterile glass bottles. Water from the Santa Engracia and El Cautivo springs was collected using a Niskin bottle (Aquatic BioTechnology, El Puerto de Santa María, Spain) both at 2 m depth. Groundwater from the S8 piezometer (60 m depth) was collected using a manual bailer system (Eijkelkamp, Giesbeek, The Netherlands). At all sampling sites, water temperature, pH, and electrical conductivity were measured with a Combo tester (Hanna Instruments, Eibar, Spain) and salinity with a density hydrometer (Hartwig Instruments, Netherlands), all parameters measured in situ. The samples were then transported to the laboratory in properly labeled containers at 4 °C and within 1 h for further processing.

### Prokaryotic Community and Diversity Study by 16S rRNA Gene Deep Sequencing

#### DNA Extraction and Sequencing

Water samples were enriched by filtering 5 L of water under aseptic conditions through a custom-made device driven by vacuum/compression pumps (Labbox, Premia de Dalt, Spain) and ending up in a 0.22-µm pore size filter. DNA was extracted using the Genomic Mini AX Bacteria Kit (A&A Biotechnology, Gdansk, Poland) and quantified using the QuantiFluor dsDNA System (Promega, Madison, WI, USA). The V3-V4 hypervariable region of the 16S rRNA gene was amplified [11], and the amplicons were sequenced using the paired-end method on a MiSeq Illumina platform at Biogenetics (Álava, Spain). Libraries were prepared from isolated DNA using the Nextera DNA Library prep kit (Ilumina, San Diego, CA, USA). Sequencing data from the study are available under the accession number PRJNA1115049.

#### Prokaryote Diversity and Distribution

Sequence analysis was performed using the packages and pipelines of the Quantitative Insights Into Microbial Ecology (QIIME2) program (version 2021.4) [12]. Joining of paired-end reads, sequence quality control, and feature table construction were performed by denoising with DADA2 plugin (q2-dada2) with standard parameters [13]. Taxonomic assignment of DADA2-generated amplicon sequence variants (ASVs) was performed using the Bayesian taxonomy classifier classify-sklearn with the q2-feature-classifier add-on [14] with taxonomy.py using the SILVA database v.138 [15], clustered at 99% sequence similarity.

Alpha diversity metrics (observed ASVs, Faith’s Phylogenetic Diversity, Pielou’s evenness, Shannon’s and Simpson’s diversity indexes) and beta diversity metric (Bray‐Curtis dissimilarity) were estimated using q2‐diversity after samples were rarefied to the smallest number of non-chimeric sequences of all samples tested. Statistical significance of the alpha diversity values between sampling sites was assessed using the Kruskal–Wallis *H* test. To assess the relationship of the water properties with *α*-diversity indices, Pearson’s coefficient test was carried out. For comparing the microbial community structure among samples, Bray–Curtis dissimilarity coefficient was assessed by analysis of similarities (ANOSIM) with 999 permutations. In all analyses, it was assumed a *p* < 0.05 for statistically significant differences.

An analysis of composition of microbiome (ANCOM) test was applied to assess differentially abundance genera using the q2-composition QIIME2 plugin [16]. A heatmap was performed by ggplot2 R package to visualize differences at genus-level composition between samples. Association between samples were established by the Bray–Curtis dissimilarity. Venn analysis was performed by Venn diagram software (available online at: http://bioinformatics.psb.ugent.be/webtools/Venn/, accessed on 13 October 2023) to determine common and unique prokaryotic taxa on samples. A Canonical Correspondence Analysis (CCA) was performed to correlate environmental variables with prokaryotic taxa and samples. To make the CCA, PAST 4 software package [17] was used and a Monte Carlo test with 999 unrestricted permutations was carried.

#### Functional Prediction of Prokaryotic Communities

The functional potential of the microbial community was predicted using 16S rRNA gene abundance data via Phylogenetic Investigation of Communities by Reconstruction of Unobserved States (PICRUSt2) [18]. The recommended maximum NSTI cut-off point of two was implemented by default in PICRUSt2, which excluded 1.3% of ASVs. Predicted pathways were categorized according to KEGG (Kyoto Encyclopedia of Genes and Genomes) orthology groups and compared between samples. In addition, the functional annotation of prokaryotic taxa via Functional Annotation of Prokaryotic Taxa (FAPROTAX) was carried out (http://www.ehbio.com/ImageGP/index.php/Home/Index/FAPROTAX.html) [19].

## Results

### Physico-chemical Characteristics of Waters

The physico-chemical analysis of the water samples revealed that they could be classified into two distinct categories: salty and brackish (Table 1). The table presents two sets of data on which the classification of the type of water is based: the first set comprises data obtained in situ at all sampling sites, while the second set comprises ion concentration data for sites belonging to the monitoring network. The values displayed for the latter dataset represent the mean value obtained from measurements conducted between 2017 and 2022. Detailed information is given in Supplementary Table S1. Monitoring showed that there is very little change in ion concentration over the years, thus adequately reflecting the stable character of the physico-chemical characteristics of the waters of the valley, which are considered stable over the years. They also maintain their flow rates (a total of about 3 L/s for the salty and 5 L/s for the brackish) fairly constant over time.

**Table 1:** Physico-chemical parameters of the water at the different sampling sites analyzed

Sampling sites			Physico-chemical parameters									
		Measured in situ				Measured by ionic chromatography*						
		Salinity (g/L)	Conductivity	T (°C)	pH	Cl− (mg/L)	${\text{SO}}_{4}^{2-}$(mg/L)	${\text{NO}}_{3}^{-}$(mg/L)	Na+ (mg/L)	Ca2+ (mg/L)	Mg2+ (mg/L)	K+ (mg/L)
Salty	Santa Engracia spring	200	Saturated	16	6.6	153,813	4697	nd	106,513	1860	286	519
	Santa Engracia channel	200	NA	17	6.6	NA	NA	NA	NA	NA	NA	NA
	El Pico spring	205	Saturated	16	7.5	168,361	5493	nd	118,100	1823	300	567
	El Cautivo spring	220	NA	17	6.6	142,721	4475	nd	100,710	1933	283	476
	Hontana spring	210	NA	16	6.2	148,983	4778	nd	99,312	1751	266	464
	Fuenterriba spring	200	NA	20	6.5	152,187	4669	nd	102,345	1845	281	580
	Pond I	200	Saturated	18	7.8	NA	NA	NA	NA	NA	NA	NA
	Pond II	210	Saturated	27	7.5	NA	NA	NA	NA	NA	NA	NA
	Pond III	220	Saturated	29	7.6	NA	NA	NA	NA	NA	NA	NA
Brackish	San Juan stream	20	NA	13	7.4	5515	921	17	3853	391	65	19
	El Pico Dulce spring	10	NA	13	7.3	5460	939	17	3812	396	62	19
	S8 piezometer	4	NA	13	7.2	3060	2374	6	2380	673	193	21

*NA* not analyzed, *nd* not detected

*These data are obtained from the monitoring network in the valley that measures these parameters periodically. The data shown are the media from the data obtained during 2017–2022

Both salty and brackish water show a clear sodium chloride facies, a direct result of the dissolution of halite in the diapiric structure. However, it is important to note the high presence of sulfate and calcium, due to the dissolution of gypsum-anhydrite, which is comparatively higher in the brackish waters (Table 1). The water taken from the S8 piezometer sampling site has a higher sulfate content than the other brackish water (Table 1), due to its particular position in the flow scheme (Fig. 1b). It is also worth noting the high concentration of magnesium and potassium in the salty waters (Table 1). In addition, as expected, nitrates are only present in the shallower brackish waters (Table 1).

The waters of the five salty springs originate from very slow deep flows (hundreds of meters) and have a very high salinity (220–240 g/L) and relatively high temperature (16–20 °C). The two brackish waters springs have a much lower salinity (15–30 g/L) and correspond to the mixture of deep waters with shallower currents. The pH of the brackish water is close to neutral (7.2 to 7.4) while the pH of the salty water is slightly lower (6.2 to 6.6).

### Prokaryotic Diversity and Community Composition

A total of 583,454 sequences corresponding to 1801 different ASVs were obtained from 11 sampling sites. The data from the Hontana spring had to be discarded due to the low number of sequences obtained. Rarefaction curves were computed by rarefying each sample to the minimum frequency of sequences, which was 14,641 reads (Fig. S1). Diversity was calculated after normalization of the samples.

#### Prokaryotic Diversity

Alpha diversity analysis revealed that prokaryotic community richness (Observed ASVs and Chao1), quantitative community richness or diversity (Shannon and Simpson indexes), and community equality or evenness (Pielou’s evenness) varied widely among the samples (Table 2). In particular, the highest prokaryotic richness, diversity, and evenness were observed in the El Pico Dulce spring, while the lowest richness was found in the El Pico spring. In contrast, the lowest diversity and evenness were found in the San Juan stream, with values similar to those of the El Pico spring.

**Table 2:** Alpha diversity indexes calculated for the locations of the saltern analyzed in the study

Sampling site	Observed ASVs	Chao1	Simpson’s index	Shannon’s index	Pielou’s evenness
Santa Engracia spring	104	104	0.95	5.07	0.76
Santa Engracia channel	196	197	0.96	5.56	0.73
El Pico spring	69	69	0.83	3.68	0.60
El Cautivo spring	169	169	0.96	5.50	0.74
Fuenterriba spring	294	301	0.93	5.48	0.67
Pond I	120	120	0.89	4.49	0.65
Pond II	74	74	0.86	3.88	0.62
Pond III	147	147	0.96	5.59	0.78
San Juan stream	155	158	0.83	3.50	0.48
El Pico Dulce spring	541	541	1.00	8.76	0.97
S8 piezometer	284	284	0.99	7.17	0.88

*ASVs* amplicon sequence variants, *Chao1* confidence interval for richness estimator

Pearson’s coefficient test showed a statistically significant negative correlation between salinity and ASVs, and also between salinity and Chao1 diversity indexes (*p* value < 0.05).

**Fig. 2:**
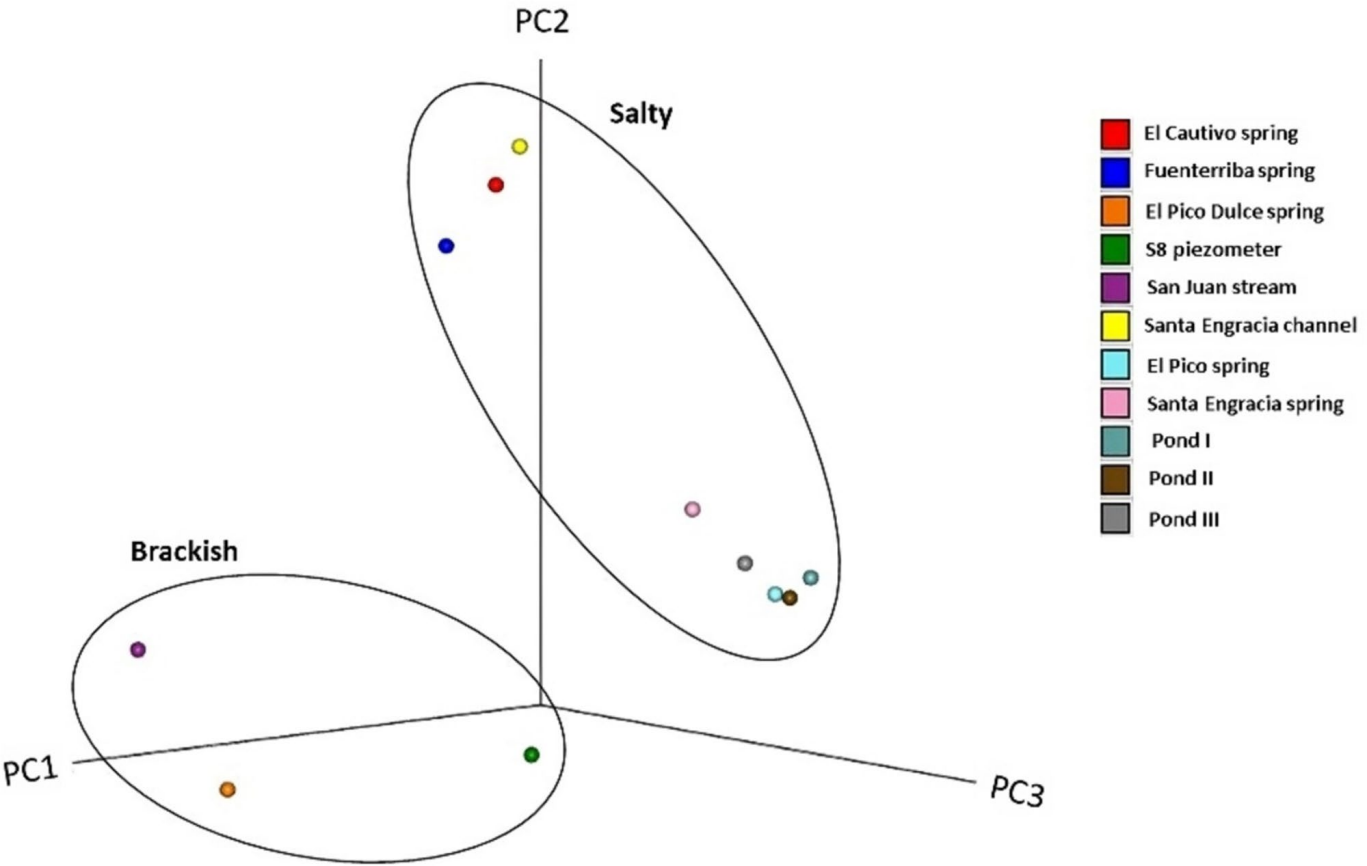
UniFrac distance-based Jackknife clustering based on the ASVs data, with the first three principal coordinates (PCs) shown: unweighted UniFrac with PC1 (26.90%), PC2 (19.21%), and PC3 (11.50%)

Beta diversity identifies dissimilarities between the sampling sites. Dissimilarity in ASV composition was represented by the Principal Coordinate Analysis (PCoA) plot. The first three principal components explained 57.61% (PC1 + PC2 + PC3) of the total variation (Fig. 2). For the analysis of multivariate homogeneity among groups, the analysis of similarities (ANOSIM) test showed significant differences in the prokaryotic diversity between groups based on their salinity (salty and brackish water) (*p*-value < 0.05). However, no statistically significant differences were observed when comparing samples according to their sampling site (spring, pond, stream, or groundwater).

#### Assessment of the Prokaryotic Community Composition and Its Distribution

**Fig. 3:**
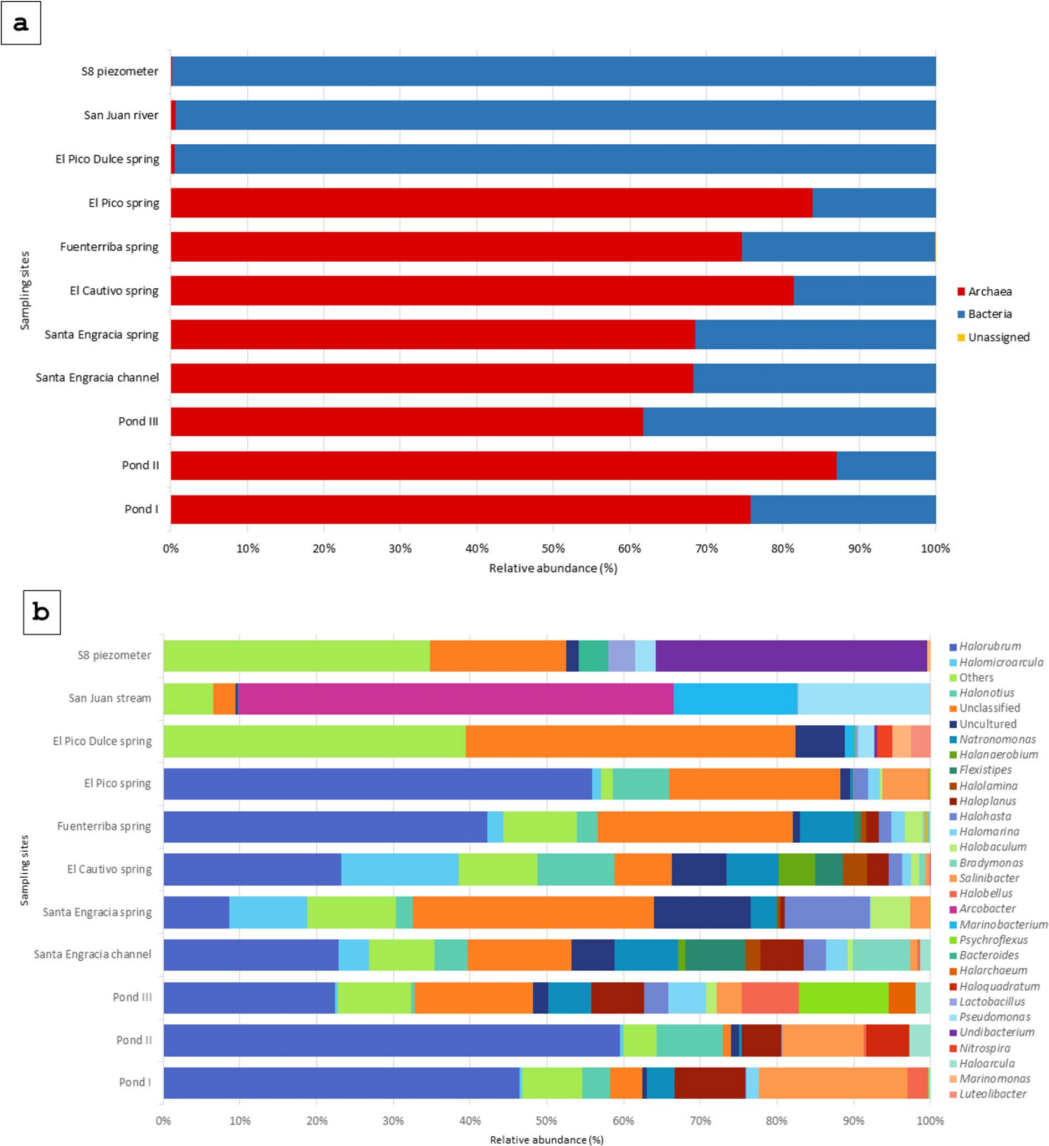
Relative abundance of the **a** domains detected in the different sample sites along the valley and **b** genera detected at different sampling sites. Genera detected at < 3% in all sampling sites are classified as Others

The 1801 different ASVs were subsequently assigned to different taxonomic levels. Fifty-nine of the ASVs (3.3%) could not be assigned to any known phylum. The taxonomic assignment of each ASV is shown in Supplementary Table S2. The taxonomic assignment showed that the archaeal domain was essentially restricted to salty waters, whereas bacteria were present at all sampling sites, with the majority in brackish water (99.4 to 99.8%) (Fig. 3a). The bacterial and archaeal domains were distributed in 31 phyla, 57 classes, 120 orders, 182 families, and 258 genera. The distribution of genera whose relative abundance was greater than 3% in at least one of the analyzed sites is shown in Fig. 3b. It can be observed that the 60.7% of the ASVs were classified at genus level (6.3% of them as uncultured organisms), while 39.3% remained Unclassified.

The major bacterial and archaeal phyla were *Pseudomonadota* and *Halobacterota*, respectively. At the genus level, the most representative bacterial genera (relative abundance > 1%) were *Pseudomonas*, *Undibacterium*, *Salinibacter*, *Marinomonas*, and *Bacteroides*. Similarly, the most abundant archaeal genera (relative abundance > 1%) were *Halorubrum*, *Halonotius*, *Haloplanus*, *Halomicroarcula*, and *Natronomonas*. Each of these genera varied greatly in abundance at different sampling sites (Fig. 3b).

**Fig. 4:**
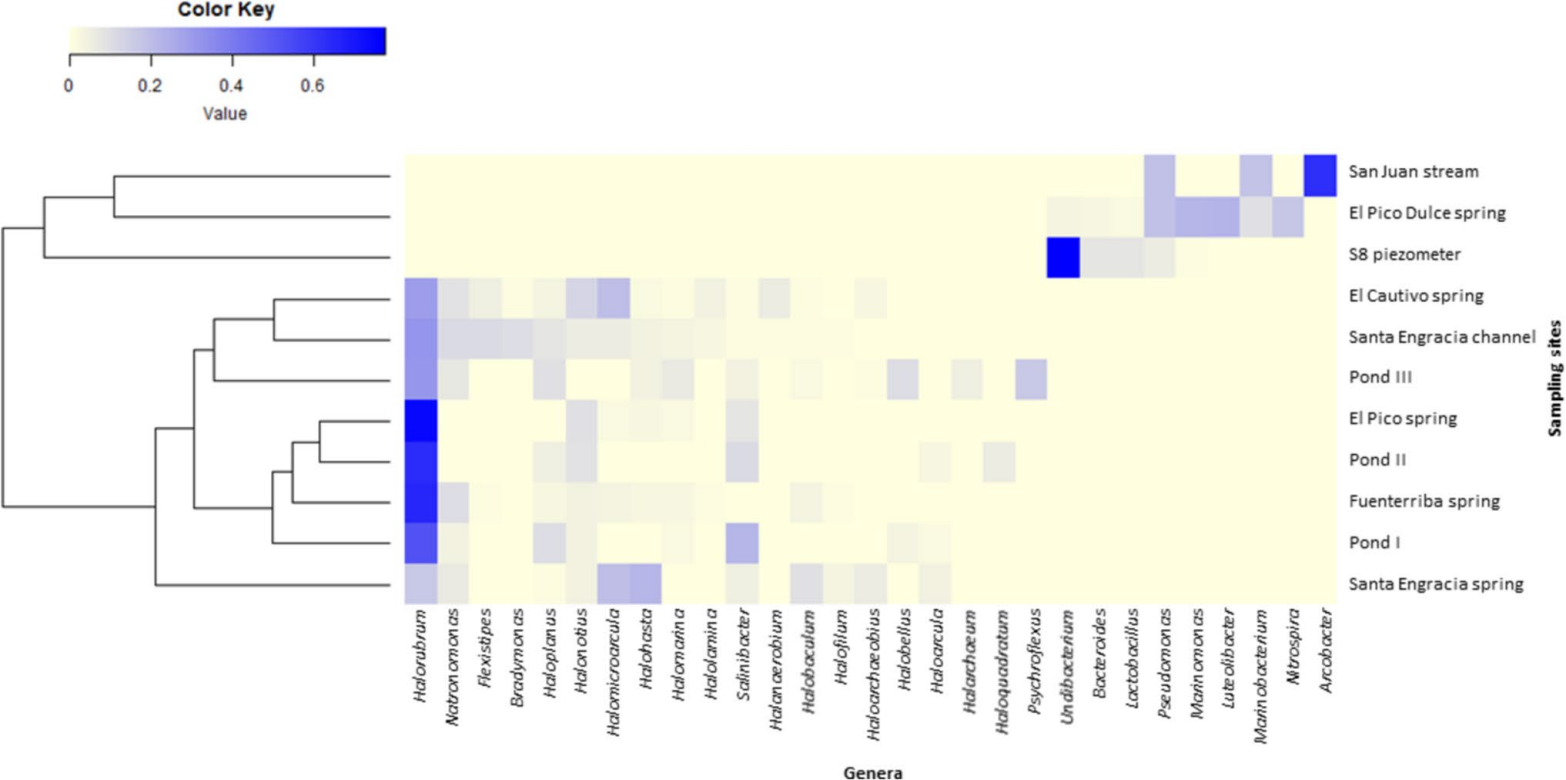
Heatmap showing the distribution of the most abundant genera (> 3% in at least one of the samples) along the sampling sites. The color represents the abundance of genera; the closer to blue, the higher the abundance, and the closer to yellow, the lower the abundance. Clustering was performed based on the Bray–Curtis dissimilarity

Although some genera were found in more than one location, different compositional profiles can be defined at the genus level. Clustering using Bray–Curtis dissimilarity coefficient based on genus abundance confirmed that brackish waters showed greater similarity to one another, as did salty waters (Fig. 4).

**Fig. 5:**
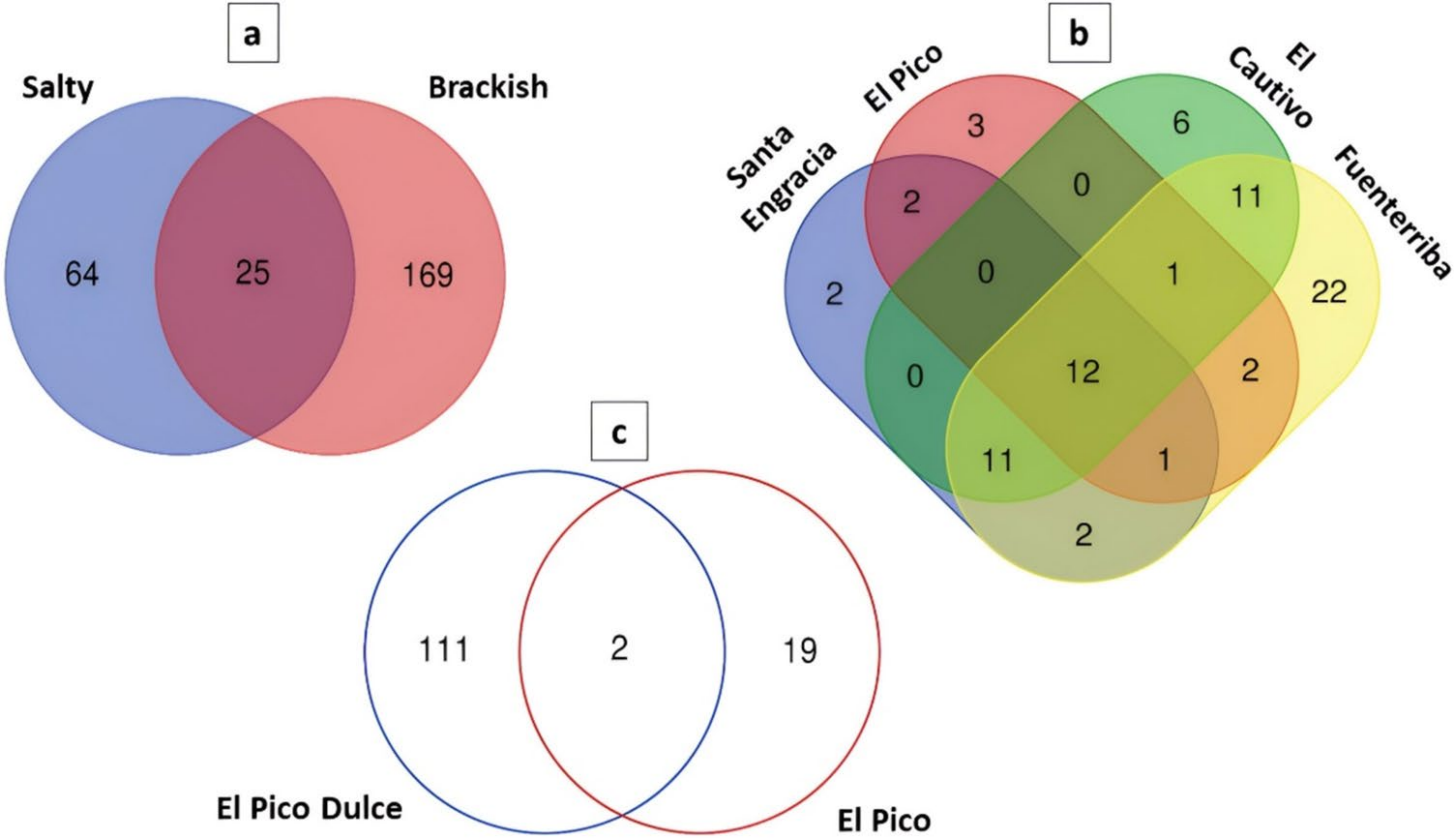
Venn plot illustrating the shared and exclusive genera between **a** the salty water and brackish water groups, **b** the four salty water springs, and **c** El Pico Dulce and El Pico springs that despite being proximal one to each other they have different water characteristics and physico-chemical composition

ANCOM analysis identified the genera *Natronomonas* and *Halorubrum* as significantly more abundant genera in the salty water samples. Furthermore, when the analysis was performed according to the water salinity of the samples, only 25 out of 258 genera were shared (Fig. 5a). However, 64 and 169 genera were unique to salty and brackish water, respectively.

The distribution of genera between salty springs revealed 12 genera shared among these samples (Fig. 5b); including *Halomarina*, *Halonotius*, *Owenweeksia*, *Halohasta*, *Natronomonas*, *Flexistipes*, *Salinibacter*, *Halorubellus*, *Haloplanus*, *Halorubrum*, *Halobaculum*, and *Halomicroarcula*. *Halorubrum* was the most abundant (8.6 to 61.2%) among them, except in the Santa Engracia spring, where *Halohasta* was the most abundant one. In the Fuenterriba spring, 22 genera were identified not present in the other saline springs. However, the genera found there were in the minority (*Acinetobacter*, 0.8%; *Marinobacter*, 0.4%; *Enterococcus*, 0.2%).

The investigation of the possibility of groundwater contact between the El Pico and the El Pico Dulce springs, which are only two meters apart but have different physico-chemical parameters (Fig. 1 and Table 1), revealed a different taxonomic composition. Of the 132 genera detected, the majority (*n* = 111) were only present at the El Pico Dulce spring and only two (*Halomonas* and *Cellulosimicrobium*) were shared (Fig. 5c). Moreover, the relative abundance of both genera based on ASV taxonomic assignment was very low in both sampling sites, 0.05% and 0.71% and 0.19% and 0.54%, respectively.

### Relationship Between Microbial Communities and Water Characteristics

The CCA results revealed the relationship between microbial community structure and water characteristics (Fig. S2). CCA axes 1 and 2 explained 77.8% of the total variance of all sampling sites. The highest values of magnesium (Mg^2+^), sulfate (${\text{SO}}_{4}^{2-}$), calcium (Ca^2+^), sodium (Na^+^), chloride (Cl^−^), and potassium (K^+^) were associated with the four salty water springs and with the presence of mainly halophilic archaea and bacteria such as *Halorubrum* and *Salinibacter*, respectively. On the other hand, the San Juan stream and El Pico Dulce spring contained the highest nitrate (${\text{NO}}_{3}^{-}$) concentration (Table 1). The genera more positively affected by these conditions appeared to be *Pseudomonas*, *Marinomonas*, *Luteolibacter*, *Nitrospira*, *Marinobacterium*, and *Arcobacter*. The particular physico-chemical characteristics of the groundwater obtained from the piezometer (Table 1) appear to favor the presence of genera such as *Undibacterium*, *Bacteroides*, and *Lactobacillus*.

Spearman's correlation test (at genus level) showed correlations between environmental variables. Thus, the only reliable statistically significant (*p* < 0.05) positive correlation was confirmed between *Halolamina*, *Halanaerobium*, *Halofilum*, and *Haloarchaeobius* (all found in saline waters) and Ca^2+^ ions.

### Prediction of the Ecological Function of Prokaryotic Microorganisms

Based on the taxonomically derived metabolic inferences, the PICRUSt2 results showed 2904 enzyme counts (ECs), 489 MetaCyc pathways, and 10,493 KEGG orthologs (KOs) belonging to 291 KEGG pathways. The metabolic MetaCyc pathways predicted by PICRUSt2 showed changes between brackish and salty water samples (Fig. S3). According to the ASV-based metabolic inference performed, L-glutamate and L-glutamine biosynthesis and pyruvate fermentation to propanoate I pathways were found with a significantly higher relative abundance in salty sampling sites, whereas toluene degradation III (aerobic, via p-cresol) and phenylacetate degradation I (aerobic) pathways were detected with a higher relative abundance in brackish water samples (Fig. S3).

**Fig. 6:**
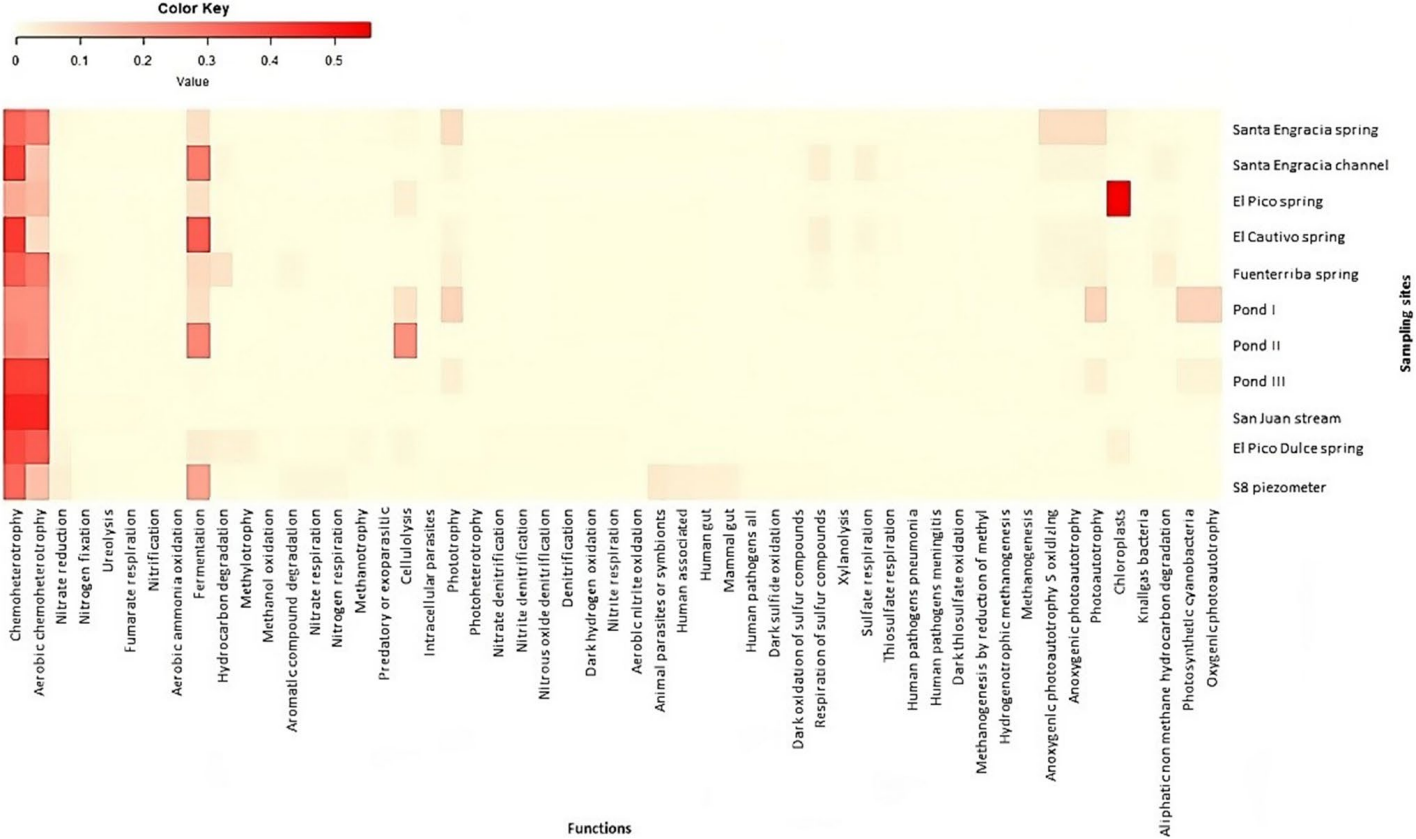
Heatmap showing the distribution of FAPROTAX predicted functions based on taxonomic assignment along the sampling sites. The color represents the abundance of microbiome involved in each ecological function; the closer to red, the higher the abundance, and the closer to yellow, the lower the abundance

FAPROTAX predicted 56 different ecological functions in at least one sampling site for bacterial and archaeal taxa derived from 16S rRNA gene amplicon sequencing (Fig. 6). The microbiome involved in chemoheterotrophy and aerobic chemoheterotrophy followed by fermentation was the one that was most widely distributed along the valley. Salty water samples had the most abundant prokaryotic groups involved in phototrophy and photoautotrophy. In particular, sulfur-oxidizing anoxygenic photoautotrophy was observed in all salty springs except in the El Pico spring, where microbiome associated with chloroplasts was predominated. The abundance of microbiome involved in oxygenic photoautotrophy and photosynthetic cyanobacteria was also observed in the ponds, except in pond II where cellulolysis was more abundant. Regarding the brackish water samples, microbiome related to animal parasites or symbionts and human pathogens was detected in the groundwater, while the abundance of microbiome involved in nitrogen metabolism was more pronounced in the San Juan stream and El Pico Dulce spring.

## Discussion

### Prokaryotic Diversity in Different Types of Water in the Valley

The Añana Salt Valley is a continental solar saltern with a thalassohaline composition. Physico-chemical monitoring of the main watercourses that feed the valley has revealed the presence of salty and brackish waters of different origins. This may be attributed to the existence of diverse pathways for unsaturated water to infiltrate the salt deposits within the subsoil, where salt dissolution occurs. [20]. This process ultimately gives rise to the emergence of springs, characterized by the emanation of water with varying salinities as a consequence of the salinization of subterranean waters. Consequently, it can be assumed that the different water flows through the Añana diapiric structure are responsible for the observed differences in the hydrochemical characteristics of the spring water.

The dissolution of evaporite (halite) hundreds of meters deep in the diapir structure is characterized by a stable ionic composition such as Cl^−^, Na^+^, and K^+^, observed in salty waters. On the other hand, the dissolution of gypsum and anhydrite, and of ${\text{NO}}_{3}^{-}$ ions, are the result of mixing of deep flows with shallower flows, characterizing the brackish waters, with comparatively higher presence of ${\text{SO}}_{4}^{2-},$ Ca^2^,^+^ and Mg^2+^ ions. The low variability of the salt facies is a peculiarity of this valley and indicates a stable depositional environment, contrary to what has been described in other diapiric environments (e.g., Zechstein 2, Germany) [20].

The differences in the physico-chemical composition of the waters showed that the main factor influencing the prokaryotic distribution in this valley was the salinity, as shown by the beta diversity indices. In fact, this microbial distribution did not appear to be influenced by parameters such as pH, temperature or even the site itself. This fact was also observed in the studies by Lozupone and Knight [21] and Leoni et al. [22]. Regarding alpha diversity indices, it was shown that samples with lower salinity had higher values of those indices than the more salty ones, as in other locations such as The Arava Valley (between the Dead Sea to the Red Sea, Israel) [23] and Saltern of Margherita di Savoia (Italy) [22]. In fact, the El Pico Dulce spring (10 g/L salinity) has the highest values of richness, diversity and evenness. On the contrary, the El Pico spring (205 g/L salinity, sited only two meters away from the El Pico Dulce spring) had the lowest richness values. This could be partially explained due to the higher salty stress experienced by microorganisms living in high salinity environments, which may have limited diversity due to the energetically costly lifestyle [24]. However, other aspects, such as the availability of nutrients and oxygen, which were not determined in this study, must be considered among the main limiting factors of microbial diversity in waters coming from very deep flows [25], which is the case of the water of this spring. Interestingly, the lowest diversity and evenness was found in the San Juan stream (brackish water), even though the measured physico-chemical characteristics of the water in this sampling site were similar to those of the El Pico Dulce spring. This suggested the presence of dominant taxa in the San Juan stream, *Arcobacter* genus in this case. The location characteristics of the sampling place result in stream water remaining in contact with the surface for a longer period than El Pico Dulce spring water. This allows for interaction with surrounding factors over a longer period (e.g., flora and rocks). This fact could provide an opportunity to certain microorganisms present in the surface areas to significantly increase their population and become dominant. In this case, the adjacent vegetation could provide an opportunity for the *Arcobacter* genus to increase its population and become dominant due to a larger surface area for adhesion [26], which could favor the formation of biofilms, an ability described in this genus [27]. In addition, a microcosm experiment in which nitrogen was added revealed a significant decline in OTU (operational taxonomic unit) richness, accompanied by a notable increase in the prevalence of the genus *Arcobacter* [28]. Given that *Arcobacter* is known to oxidize sulfur as well as reduce nitrate, the authors propose that sulfur may serve as an electron donor in denitrification but may also inhibit the reduction of nitrous oxide to nitrogen gas. This highly specific niche may account for the pronounced reduction in species richness observed in the experiment, potentially linked to trace element accessibility. It is plausible that a similar scenario is occurring in the San Juan stream.

### Community Composition and Distribution

The taxonomic approach performed in this study showed that the salty waters of this saltern were essentially restricted to the domain of *Archaea*. However, bacteria were present in all sampling sites and were particularly abundant in brackish waters. This distribution has been previously described in other hypersaline environments [22, 23, 28], confirming that the salinity shapes the microbial community. Furthermore, it was found that the main phyla found in the valley were *Pseudomonadota* and *Halobacteriota*. The archaeal phylum *Halobacteriota* is the best known group of extreme halophiles [6], and the predominance of the *Pseudomonadota* phylum in hypersaline environments has also been observed [5]. The study of the prokaryotic community by Illumina-based 16S rRNA gene sequencing in hypersaline environments reveals the possibilities and limits of life in the most extreme conditions [29], bearing in mind that it is based on the putative association of the 16S rRNA gene with a taxon defined as an amplicon sequence variant (ASV).

The genus-level study of the main waters supplying the valley (springs, the stream, and the groundwater), identified two main distinct microbial communities, one from brackish waters and the other from salty waters. Even if all the salty waters are considered to have the same origin, there are marked differences in the relative abundance of the ASVs belonging to the main genera, with some of them accounting for less than 1% of the relative abundance in some sampling sites. Thus, a niche effect can also be observed, but with less influence than salinity. There is a study of hypersaline soils where site-specific characteristics correlated with community structure and salinity played a secondary role [30], which is not the case in the Añana Salt Valley. Within the brackish waters, according to the CCA analysis, which relates physico-chemical data to community structure, a subdivision was observed. Specifically, groundwater obtained from the piezometer was observed to exhibit distinct characteristics compared to the other brackish waters. The physico-chemical variables studied explained about 78.6% of the variance in community structure between samples. No specific water physico-chemical variables predicting community structure could be identified. However, it was observed a higher relative abundance of the genera *Halolamina*, *Halanaerobium*, *Halofilum*, and *Haloarchaeobius* when the Ca^2+^ ion concentration was higher. All but *Halophilum* belonged to the *Nanohaloarchaeota*, in agreement with Vera-Gargallo et al. [30] who also detected *Nanohaloarchaeota* associated with Ca^2^⁺ ions.

Regarding the salty waters community, the most abundant archaeal ASVs belonged to the genus *Halorubrum*, followed by the genus *Natronomonas*, except in the Santa Engracia spring, where *Halohasta* was the most abundant one. Members of the genus *Halorubrum* have also been found in high abundance in the few studies carried out using 16S rRNA gene sequencing in continental salterns; at Redonda and Penalva inland salterns in Spain [31] and at Ciechocinek Spa on a salt diapir in Poland [32]. However, its abundance in coastal salterns, such as the Santa Pola salt marshes [25], is of secondary occurrence. Abundance of the genus *Halohasta* (in this study mainly in the Santa Engracia spring, which is the main brine supplier of the saltern) was also reported in water samples from the Ciechocinek Spa. The authors reported that the genus *Halohasta* was part of a stable community together with *Natronomonas*, *Halobacterium*, and *Haloplanus* in waters with salinities between 16 and 27% [32]. On the other hand, the genus *Pseudomonas* is also an important part of the prokaryotic community inhabiting the Añana’s saltern, being present in all the brackish waters, although in different abundances. The genus *Pseudomonas* has previously been found in hypersaline environments [33] due to its versatile energetic metabolism, which allows it to live in different environments. Finally, the differentiation of the groundwater obtained from the piezometer from the other brackish waters was due to the high relative concentration of sulfate and the high abundance of the genus *Undibacterium*. These two factors may be related, as seen in the study by Damo et al. [34] in which an increase of the genus *Undibacterium* was observed in the rhizosphere where sulfur (oxidized to available sulfate by microbes) was applied.

When comparing the relative abundance of the genera in the salty waters involved in the salt production system (the channel and brine resting ponds), a decrease in the genera *Halohasta* and *Halomicroarcula* was observed, which were abundant in the Santa Engracia spring. The *Halomicroarcula* genus appeared to be exclusively to groundwater brine samples, as seen in Najjari et al. [35], where they did not found *Halomicroarcula* in the sediment or halite crusted samples at the same sampling site. Once in the resting ponds, the relative abundance of the genera *Haloplanus* and *Salinibacter* increased in comparison to the salty springs waters. However, it should be noted that the water from different salty springs could be mixed in these ponds, although water from Santa Engracia is the more abundant, as previously stated. The genus *Salinibacter* is the bacterium that can be found even in the saltiest waters; in the case of Margherita di Savoia (Italy), it reaches its highest abundance in saline ponds with a salinity of 30.6% [22]. In addition, a number of species of the genus *Haloplanus* have even been isolated from commercial salt crystals [36]. These results may indicate that some abundant microbial genera found in the resting ponds may play a role in the salt production from the Añana Salt Valley, although their importance remains unknown.

Lastly, the high percentage of ASVs assigned to Unclassified genera (39.3%), especially in the El Pico Dulce spring (43.0%), together with the large number of taxa grouped as Uncultured (12.6%) in the Santa Engracia spring, was striking in this study. This could mean that they correspond to sequences of taxa (genera or species) that have not yet been identified. Culture-based microbiological studies carried out in this saltern support this hypothesis [37, 38]. This could mean that dominant or abundant taxa could be overtaken by new unidentified taxa, as mentioned by Oren 2006 [1].

### Prediction of Microbial Functions

According to the metabolic prediction performed based on the 16S rRNA gene datasets on this study, the significant increase in pyruvate fermentation to propionate I found in the salty water samples is striking, as fermentative halophilic archaea are very rare [39]. However, some genera of halophilic archaea, such as *Halorubrum* or *Haloarcula*, which are largely found in the salty water samples of this study, have the ability to convert sugars to pyruvate [40]. It was shown in *Halobacterium salinarum* that pyruvate can be utilized under anaerobic conditions [41], although the understanding of pyruvate transport in halophilic archaea is very limited [40]. However, it is important to acknowledge that, despite the established efficacy of metabolic inference based on 16S rRNA gene datasets, this technique is not without its inherent limitations. These include the availability of correct published sequenced genomes and high genomic plasticity [42].

Taxonomic-inferred metabolic prediction showed that the microbiome involved in chemoheterotrophy and fermentation was the most abundant and widespread along the valley. It is known that most halophilic prokaryotes are aerobic chemoorganotrophs, capable of degrading organic compounds up to NaCl saturation [43]. However, oxygen is poorly soluble in brines, allowing anaerobic halophilic heterotrophs to thrive [43] and actively coexist in the same niche. According to Wang and Bao [5], chemoheterotrophy is the main bacterial function, but also predominate in archaea, while fermentation is widespread among bacterial functions.

The present study also found that salty water samples, dominated by archaea, had taxa involved in phototrophy. Many haloarchaea are known to have the ability to grow phototrophically, making them physiologically versatile microorganisms [44]. Chloroplast function detected in El Pico spring was also noteworthy. It seems plausible to suggest that the detection of chloroplast function is related to the presence of cyanobacteria, due to the similarity between the genetic material of chloroplasts and that of certain bacteria [45]. Therefore, errors in taxonomical assignment may occur [46]. This phenomenon was observed in certain instances in the taxonomic assignment, with some sequences, identified as cyanobacteria at phylum level, being erroneously assigned as chloroplasts. Consequently, as the identification of metabolic functions is based on taxonomic assignment in this study, this inaccuracy can also be extrapolated to the determination of functions.

Microbiota involved in nitrogen metabolism was also found, especially in brackish water from San Juan stream and El Pico Dulce, with the genus *Pseudomonas* found to be widespread. There is a study in which *Pseudomonas* was the dominant genus in epiphytic bacterial communities, suggesting that the epiphytic bacteria of submerged plants may play an important role in the denitrification [47]. This may be the case at these two sampling sites, which are surrounded by local vegetation and whose waters are influenced by shallower water flows with the presence of nitrogenous compounds from agricultural practices. Lastly, water from the S8 piezometer sampling site showed major abundance of microbiota involved in animal parasites or symbionts and human pathogenicity due to the presence of *Bacteroides* and *Lactobacillus*, both genera related to the gut microbiota [48]. This fact supports the idea of the presence of lateral flow waters, without an upward component, coming from shallower areas to the east of the diapiric structure, where several livestock farms are located. These findings on the putative ecological functions of the prokaryotic community could be considered as a further piece of evidence to support the approach to the origin of the water samples studied in this work.

## Conclusions

The study of the prokaryote community and its distribution, together with the physico-chemical analysis of the main waters supplying the valley, distinguishes salty and brackish waters. Archaea were mainly restricted to salty water, whereas bacteria were present in all sampling sites. Considering all the salty spring waters of the same origin, given their physico-chemical similarities, the differences observed at the prokaryotic genus level could be due to site-specific characteristics, suggesting the enormous and still unknown importance of niche specificity. However, our results do support the hypothesis that the origin of the water and the associated salinity stress affect the microbiome beyond the niche location in the valley. This is evident in the El Pico and El Pico Dulce springs, located just two meters from each other in the valley, that have different water origin and different microbial compositions. Further studies are needed, including more groundwater samples from other piezometers located in the valley, to gain insights into water flow scheme, together with physiology and ecology studies of microbial taxa and isolation of new undescribed microorganisms.

## Supplementary Information

Below is the link to the electronic supplementary material.

Supplementary file1 (PDF 496 KB)

Supplementary file2 (XLSX 219 KB)

## Data Availability

Sequencing data from the study have been deposited in the GenBank under the accession number PRJNA1115049.
