# Supplementary material for: Prokaryotic Diversity and Community Distribution in the Complex Hydrogeological System of the Añana Continental Saltern
Source: Microb Ecol. 2025 Jan 16;87:171. doi: 10.1007/s00248-025-02488-2 (PMC11739210; doi:10.1007/s00248-025-02488-2)
Supplement: Supplementary file 1 [file 248_2025_2488_MOESM1_ESM.pdf]

Supplementary material

Prokaryotic diversity and community distribution in the complex hydrogeological system of the Añana continental saltern

Maia Azpiazu-Muniozguren[a], Minerva García-Martínez[a], Ane Zabaleta[b], Iñaki Antiguedad[b], Javier Garaizar[a,c], Lorena Laorden[a,c], Irati Martinez-Malaxetxebarria[a,c], Ilargi Martinez-Ballesteros[a,c*]

[a]*MikroIker Research Group, Immunology, Microbiology and Parasitology Department, Faculty of Pharmacy, University of the Basque Country UPV/EHU, Paseo de la Universidad 7, 01006, Vitoria-Gasteiz, Spain.*

[b]*Hydro-Environment Processes Research Group. Geology Department, Faculty of Science and Technology, University of the Basque Country UPV/EHU, Barrio Sarriena s/n, 48940, Leioa, Spain.*

[c]*Bioaraba, Microbiology, Infectious Diseases, Antimicrobial Agents, and Gene Therapy, 01006, Vitoria-Gasteiz, Spain.*

*Corresponding author.

E-mail address: ilargi.martinez@ehu.eus (I. Martinez-Ballesteros)

MATERIAL AND METHODS

Geological and hydrogeological context

The Añana Salt Valley is located to the northwest of the Añana diapir, an ellipsoidal structure of 13 km^2 with an E-W extension. The origin of the salt is a large evaporite rock formation (mainly halite and gypsum) formed when the Sea of Tethys was subjected to very strong evaporation at the end of the Triassic period, just over 200 million years ago. On this salty formation, marine and continental formations (limestones, dolomites, sandstones, marls, siltstones, clays, and volcanic ophites) were deposited throughout the Miocene, according to the different environmental conditions. Subsequently, important tectonic forces used the salty formation as a plane of detachment, creating a diapiric structure in which the salty materials from the bottom (about 5 km deep), which are less dense, rise and break the upper formations until they reach the surface. Unlike other nearby diapiric structures, the Añana structure, which is still active, has resulted in a complex mosaic of different rocks in both shallow and deep zones.

This geological complexity leads to hydrogeological complexity, as the different rocks contained in the salt mass have different degrees of permeability. As the salts rise, they are dissolved by groundwater flows, creating salty and brackish water springs along the valley (Fig. 1a). The waters of the five salty springs come from very slow deep flows (hundreds of meters) and have a very high salinity (220-240 g/L) and relatively high temperature (16-20 °C); these are the waters used for salt production. The two brackish waters have a much lower salinity (15-30 g/L) and correspond to the mixture of deep waters with shallower currents.

All waters, both salty and brackish waters, maintain their physico-chemical characteristics (Table 1) and flow rates (a total of about 3 L/s for the salty and 5 L/s for the brackish) fairly constant over time as a result of a highly temporally regulated groundwater flow regime. The time lag between rainwater recharge and discharge in salty springs can exceed a dozen years, according to initial isotopic analyses.

Sampling sites features

The Santa Engracia spring is situated at the highest point of the valley and it is the main brine supplier to the saltern. Immediately, its water is channeled (called in this study Santa Engracia channel) and distributed throughout the valley. This salty water spring is very close to the brackish water source of the San Juan stream, which runs all along the saltern. Downstream from the main spring are the other springs, some with salty water (El Cautivo, El Pico, Fuenterriba, Hontana) and some with brackish water (El Pico Dulce, which is only 2 meters from El Pico). To the east, in the valley, there is a piezometer (S8) from which brackish groundwater can be sampled at a depth of 60 meters. Finally, in the northern part are the three ponds (Pond I, Pond II and Pond III, for the temporary accumulation of the salt water) at the end of the salt production system.

FIGURES

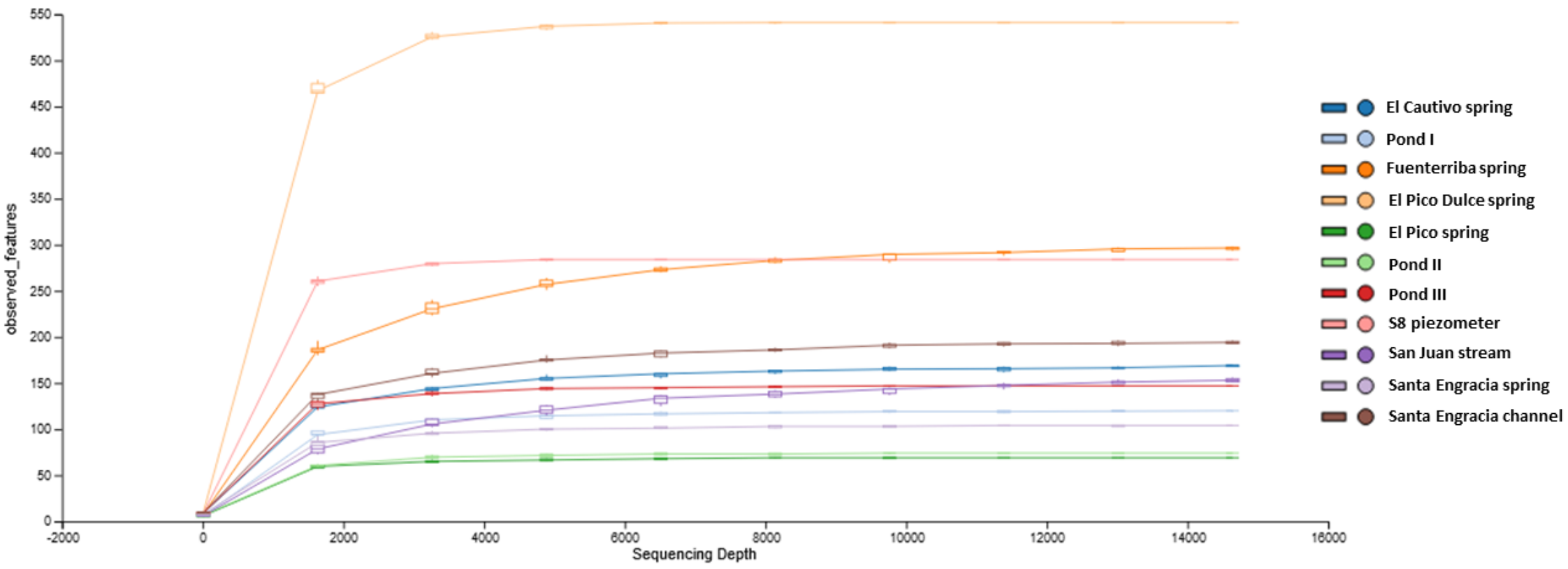

Fig. S1 Rarefaction curves computed thought QIIME2 depicting distribution and diversity of microbial communities in each sampling site using observed ASVs at 99% sequence similarity.

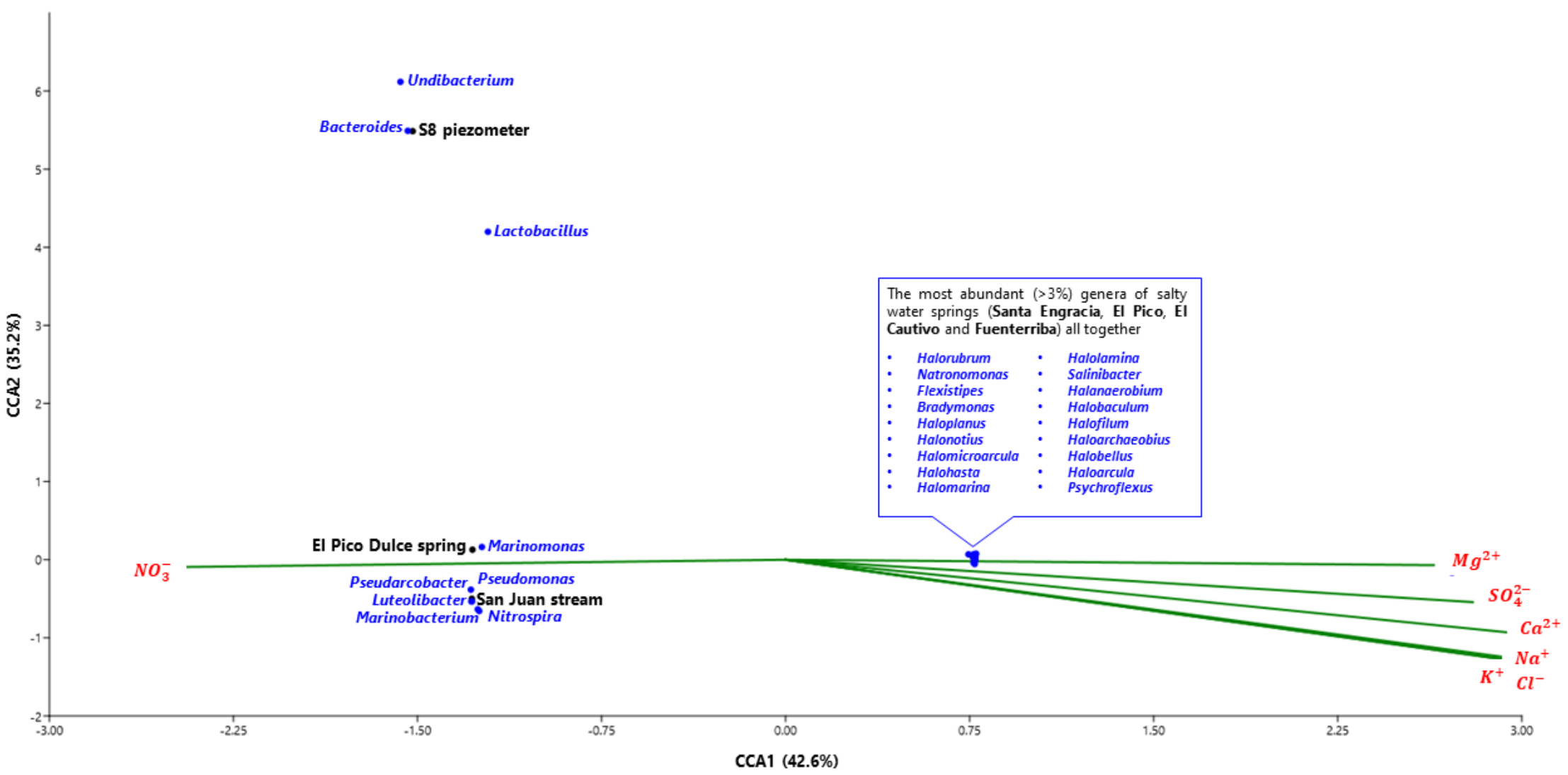

Fig. S2 CCA analysis performed on water from S8 piezometer, El Pico Dulce spring, San Juan stream, El Cautivo spring, Santa Engracia spring, Fuenterriba spring and El Pico spring. Ordination diagram of the prokaryotic genera and physico-chemical data according to axis 1 and 2. Green lines represent quantitative environmental variables indicating their direction of increase. NO_3^-, nitrate; Mg^{2+}, magnesium; SO_4^{2-}, sulphate; Ca^{2+}, calcium; Na^+, sodium; Cl^-, chloride; K^+, potassium.

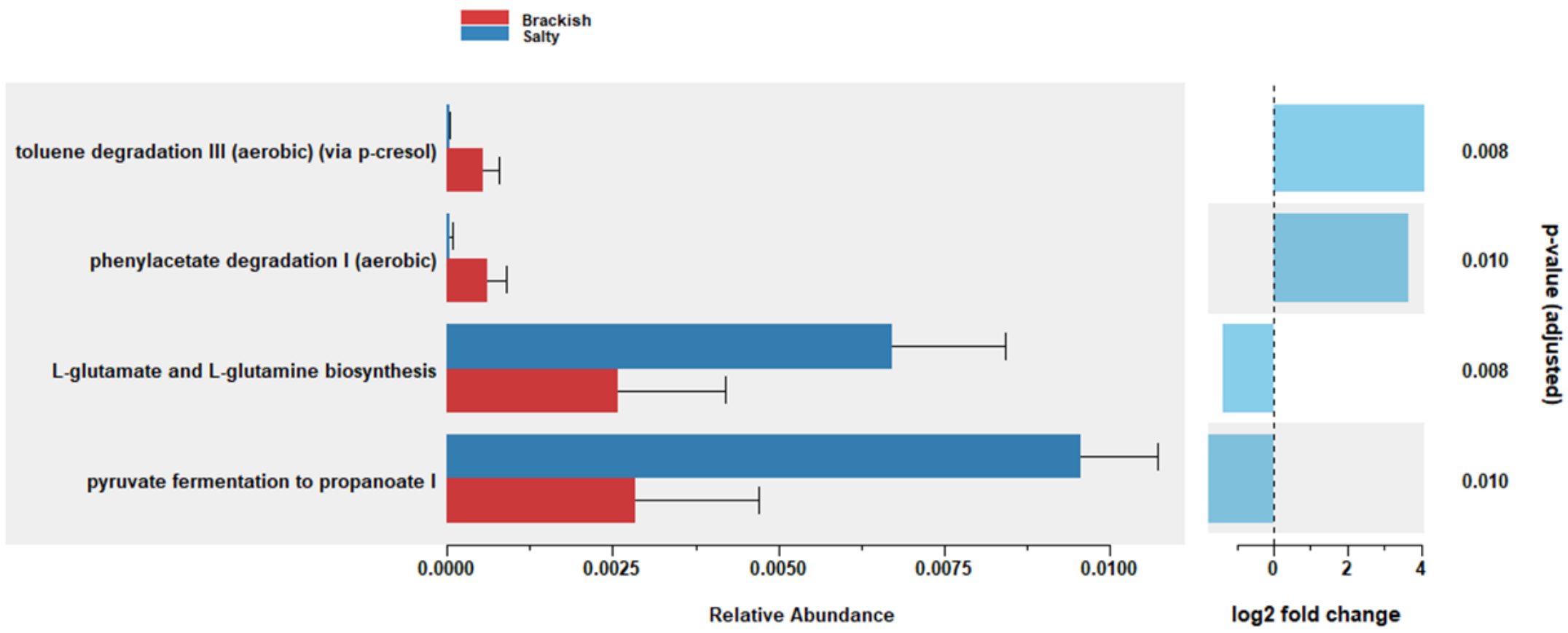

Fig. S3 Significantly different MetaCyc annotated metabolic pathways predicted by PICRUSt2 between brackish and saline water samples.

Table S1. Ion concentration data for points belonging to the monitoring network in the valley. The mean value obtained from measurements conducted between 2017 and 2022 is showed along with the standard deviation.

Sampling sites	Physico-chemical parameters						
	Measured by ionic chromatography						
	Cl^- (mg/L)	SO_4^{2-}(mg/L)	NO_3^- (mg/L)	Na^+ (mg/L)	Ca^{2+} (mg/L)	Mg^{2+} (mg/L)	K^+ (mg/L)
**Santa Engracia spring**							
number of samples	11	11	11	9	10	9	9
arithmetic mean	153812,6	4696,8	nd	106513,0	1859,6	286,1	518,5
standard deviation	19286,1	331,9		6372,6	189,0	35,5	37,7
**El Pico spring**							
number of samples	2	2	2	1	1	1	1
arithmetic mean	168361,0	5492,5	nd	118100,0	1823,0	300,0	567,0
standard deviation	39783,2	953,9					
**El Cautivo spring**							
number of samples	4	4	4	4	4	4	4
arithmetic mean	142721,3	4475,3	nd	100709,5	1933,3	282,8	476,2
standard deviation	9290,0	236,7		7661,8	185,1	46,3	38,7
**Hontana spring**							
number of samples	3	3	3	2	3	2	2
arithmetic mean	148983,0	4778,0	nd	99311,5	1751,0	265,7	463,7
standard deviation	1744,5	142,8		9904,4	9,5	21,6	124,9
**Fuenterriba spring**							
number of samples	5	5	5	3	5	3	3
arithmetic mean	152187,4	4668,8	nd	102344,7	1844,6	281,1	579,7
standard deviation	21419,8	176,4		5747,1	266,4	6,2	24,9

**San Juan stream**							
number of samples	10	10	10	8	9	8	8
arithmetic mean	5293,7	920,5	17,2	3876,0	390,6	65,2	18,7
standard deviation	1406,5	85,1	2,7	757,3	27,5	1,8	3,0
**El Pico Dulce spring**							
Number of samples	12	12	12	9	11	9	9
arithmetic mean	5270,0	938,6	16,9	3881,3	396,5	62,1	18,7
standard deviation	1351,6	90,6	2,6	1036,1	30,8	7,2	4,0
**S8 piezometer**							
number of samples	7	7	7	5	7	5	5
arithmetic mean	2833,7	2374,4	6,2	2469,4	672,7	193,2	20,7
standard deviation	1160,3	618,4	4,7	606,5	83,8	46,5	13,0

nd: not detected
